# Host–guest complexes of mixed glycol-bipyridine cryptands: prediction of ion selectivity by quantum chemical calculations, part V

**DOI:** 10.3762/bjoc.9.142

**Published:** 2013-06-27

**Authors:** Svetlana Begel, Ralph Puchta, Rudi van Eldik

**Affiliations:** 1 Inorganic Chemistry, Department of Chemistry and Pharmacy, University of Erlangen-Nürnberg, Egerlandstr. 1, 91058 Erlangen, Germany; 2 Computer Chemistry Center, Department of Chemistry and Pharmacy, University of Erlangen-Nürnberg, Nägelsbachstr. 25, 91052 Erlangen, Germany

**Keywords:** cryptands, DFT, quantum chemistry, selective ion complexation

## Abstract

The selectivity of the cryptands [2.2.bpy] and [2.bpy.bpy] for the endohedral complexation of alkali, alkaline-earth and earth metal ions was predicted on the basis of the DFT (B3LYP/LANL2DZp) calculated structures and complex-formation energies. The cavity size in both cryptands lay between that for [2.2.2] and [bpy.bpy.bpy], such that the complexation of K^+^, Sr^2+^ and Tl^3+^ is most favorable. While the [2.2.bpy] is moderately larger, preferring Rb^+^ complexation and demonstrating equal priority for Sr^2+^ and Ba^2+^, the slightly smaller [2.bpy.bpy] yields more stable cryptates with Na^+^ and Ca^2+^. Although the CH_2_-units containing molecular bars fixed at the bridgehead nitrogen atoms determine the flexibility of the cryptands, the twist angles associated with the bipyridine and glycol building blocks also contribute considerably.

## Introduction

The present report continues a series of contributions from our group dealing with quantum chemical investigations of the selective complexation of alkali and alkaline-earth metal cations by supramolecular species, predominantly cryptands and their derivatives [1–4]. The current state of research relevant for our studies was carefully explored and illustrated in the mentioned publications and will therefore not be repeated in detail in this work.

Selective complexation of molecules and ions is one of the most important topics in bio-inorganic supramolecular chemistry, which requires detailed and elaborate examination due to its significant role in receptors in biological and technical systems. Selectivity phenomena have been studied for over 80 years [5–6]. During this period of time versatile model systems with variable cavity sizes have been investigated experimentally as well as by computational methods to gain information about the geometric and electronic demands of this process [7–10]. Well-known supramolecular species, e.g., calixarenes [11–15], cyclodextrines [16], crown ethers [17–22], cryptands [23–24] and the corresponding metallatopomers, easily accessible by self-organisation [25–36], can be taken as examples for such model systems.

**Figure 1:**
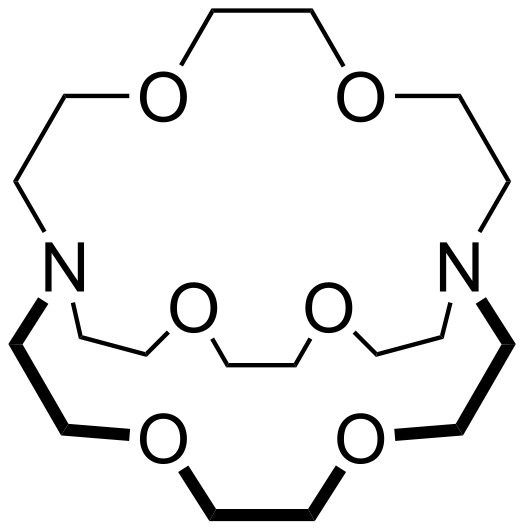
Structure of [2.2.2] also known as 4,7,13,16,21,24-hexaoxa-1,10-diazabicyclo[8.8.8]hexacosane or Kryptofix 222.

Kryptofix 222 ([2.2.2] or 4,7,13,16,21,24-hexaoxa-1,10-diazabicyclo[8.8.8]hexacosane) (Figure 1), synthesized by Lehn 40 years ago [37] is still very popular and widely used today, especially due to its ability to bind ions selectively, primarily alkali and alkaline earth metal cations [38–49]. In contrast, derivatives of this cryptand containing, for example, nitrogen donor atoms ([phen.phen.phen] and [bpy.bpy.bpy]) as well as the hybrids between them and [2.2.2] have not yet been studied sufficiently [50–58]. While the main concern was their photophysical and photochemical properties [51–52,54–56,59–60], their ability for selective complexation was not investigated experimentally.

As mentioned above, the complexation of alkali and alkaline-earth metal ions by different cryptands was investigated extensively in our group on the basis of DFT (B3LYP/LANL2DZp) calculations [1–4]. Within the framework of this study, nondynamic quantum chemical calculations, performed in the absence of solvent molecules and focusing on the system itself, were utilized very successfully for the careful examination of the supramolecules, excluding possible disturbing side effects.

**Figure 2:**
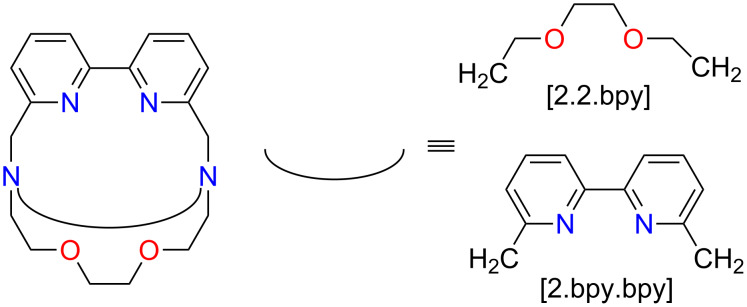
Structures of [2.2.bpy] and [2.bpy.bpy].

The current work extends our explorations into the outlined topic. Here we discuss two hybrid cryptands between [2.2.2] and [bpy.bpy.bpy]. They are abbreviated as [2.2.bpy] and [2.bpy.bpy] and are presented in Figure 2.

## Results and Discussion

**Figure 3:**
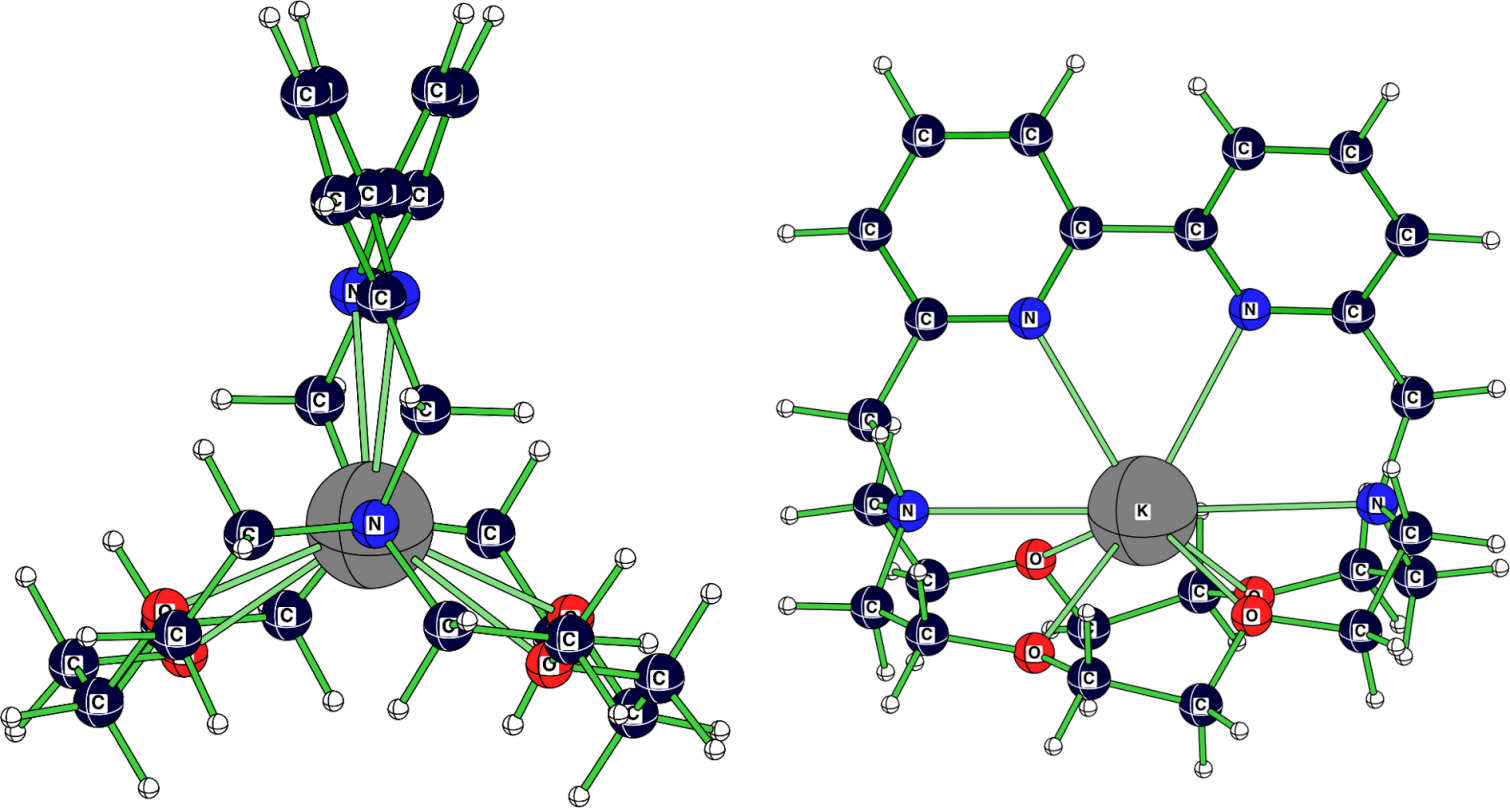
Calculated (RB3LYP/LANL2DZp) structure (*C_2_*) for [K 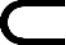 2.2.bpy]^+^.

**Figure 4:**
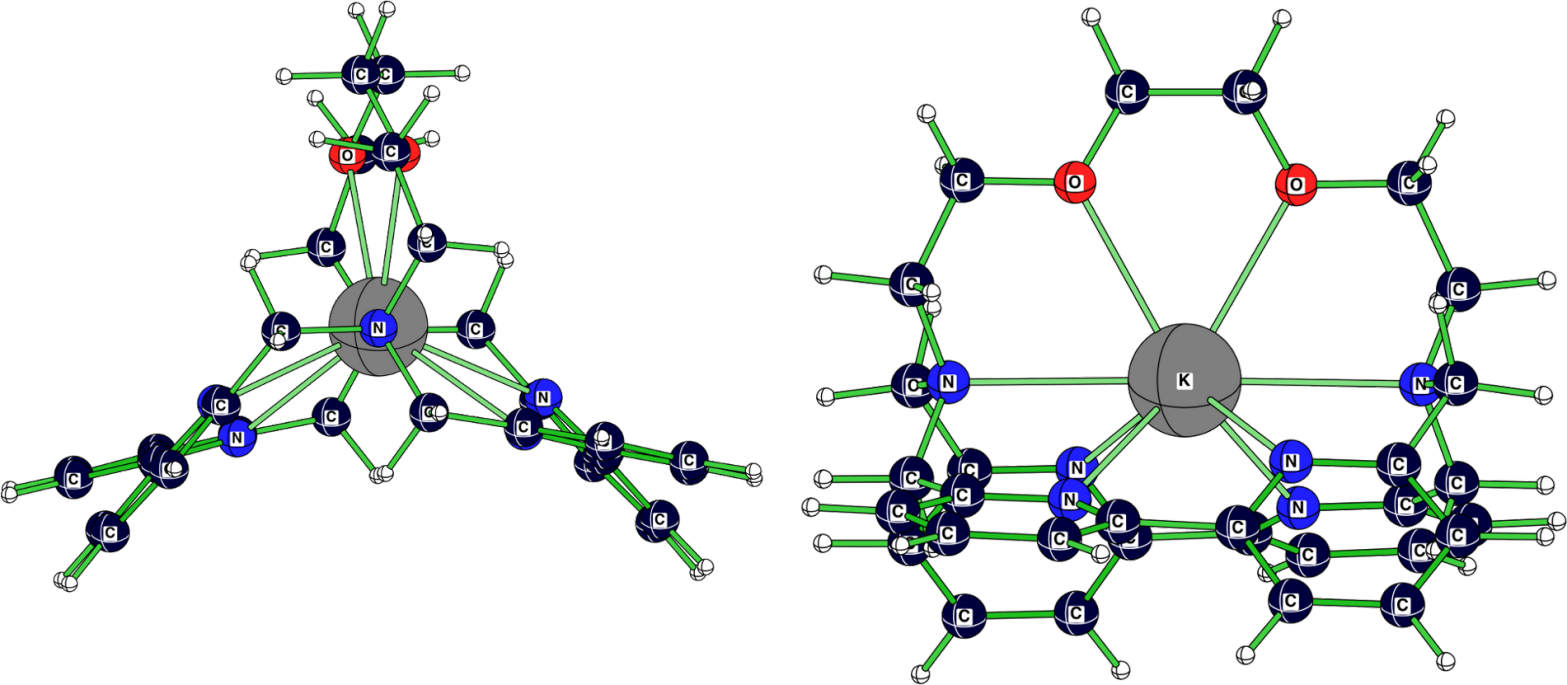
Calculated (RB3LYP/LANL2DZp) structure (*C_2_*) for [K 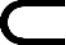 2.bpy.bpy]^+^.

Although as of spring 2013 more than 650 X-ray structures are listed in the Cambridge Structural Database for [2.2.2] and mostly its alkali and alkaline-earth metal cryptate complexes, only the structure of [Na 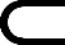 2.2.bpy]Br has been published [61], and to the best of our knowledge there are no further experimental structures for [2.2.bpy] and [2.bpy.bpy] cryptands and cryptate complexes. In earlier investigations on related supramolecular systems the applied method (RB3LYP/LANL2DZp) provided satisfactory results [1–4]. In all these cases the calculated bond length between the guest ions and the donor atoms was elongated compared to the analogous bonds in the X-ray structures. The same behavior is found when comparing [Na 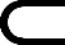 2.2.bpy]Br and the (B3LYP/LANL2DZp) calculated *C_2_* symmetric [Na 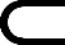 2.2.bpy]^+^-ion. The bonds between the sodium cation and the donor atoms are around 5.5% longer than in the averaged solid-state structure (Table 1). Whereas Table 1 and Table 2 present significant data for all discussed structures, Figure 3 and Figure 4 illustrate, as representative examples, the calculated [K 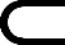 2.2.bpy]^+^ and [K 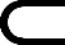 2.bpy.bpy]^+^. Table 3 and Table 4 summarize the values for the complexation energies of the studied cryptate complexes.

**Table 1:** Calculated (RB3LYP/LANL2DZp) structural data for the metal–donor interactions in [M 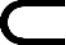 2.2.bpy]^m+^ (calculated structures: *C_2_* symmetry); (x): averaged experimental X-ray structural data [61].

	M–N_sp2_ [Å]	M–N_sp3_ [Å]	M–O [Å]	N_sp2_–C•••C–N_sp2_ [°]	CH_2_–N_sp3_•••N_sp3_–CH_2_ [°]	O–C•••C–O [°]	CH_2_–CH_2_–N_sp3_•••N_sp3_–CH_2_–CH_2_ [°]

empty	–	–	–	132.3	10.0	164.3	10.3
Li^+^ (*C_1_*)	2.24, 2.16	2.58, 2.87	3.19, 3.01, 3.07, 3.29	5.8	-92.5	56.8, 56.1	−91.0, −88.5
Li^+^	2.19	2.71	3.15, 3.12	5.0	−93.8	56.0	−91.1
Na^+^	2.79	2.90	2.84, 2.81	29.0	−67.0	59.0	−69.0
Na^+^(x)	**2.61**	**2.76**	**2.66,** **2.68**	**24.9**	−**78.6**	−**61.4**	−**77.3**
K^+^	2.95	3.03	2.89, 2.84	42.3	−44.4	64.3	−47.0
Rb^+^	3.02	3.07	2.94, 2.92	47.9	−36.2	68.5	−38.2
Cs^+^	3.12	3.12	3.02, 3.03	56.1	−28.8	76.0	−30.2
Be^2+^ (*C_1_*)	1.78, 1.66	1.83, 3.64	1.65, 3.76, 4.50, 3.92	3.0	−123.1	55.5, 76.2	−89.6, −120.1
Be^2+^	1.79	1.95	3.24, 3.65	3.2	−136.0	49.6	−120.2
Mg^2+^ (*C_1_*)	2.24, 2.23	2.47, 2.57	2.33, 2.27, 3.42, 3.45	−5.2	−113.0	50.9, 41.9	−112.7, −105.8
Mg^2+^ (T.S.)	2.29	2.48	2.71, 2.74	−7.9	−116.9	47.2	4.5
Ca^2+^	2.68	2.74	2.68, 2.68	−6.2	−92.8	49.8	−94.5
Sr^2+^	2.81	2.87	2.77, 2.76	20.0	−72.6	52.9	−74.5
Ba^2+^	2.92	2.98	2.86, 2.84	31.5	−50.1	57.0	−52.7
Al^3+^	2.07	3.13	2.02, 2.09	−24.4	−86.7	40.1	−82.1
Ga^3+^ (*C_1_*)	2.05, 2.05	2.22, 2.20	2.11, 2.16, 3.76, 3.80	−0.3	−127.1	48.0, −38.4	−113.1, −6.7
Ga^3+^ (T.S.)	2.06	2.14	2.86, 3.01	−0.7	−130.8	41.4	−125.6
In^3+^	2.31	2.48	2.46, 2.47	−12.4	−126.3	42.3	−127.1
Tl^3+^	2.48	2.55	2.71, 2.73	−6.2	−111.2	49.1	−111.6

**Table 2:** Calculated (RB3LYP/LANL2DZp) structural data for the metal–donor interactions in [M 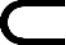 2.bpy.bpy]^m+^ (calculated structures: *C_2_* symmetry).

	M–N_sp2_ [Å]	M–N_sp3_ [Å]	M–O [Å]	N_sp2_–C•••C–N_sp2_ [°]	CH_2_–N_sp3_•••N_sp3_–CH_2_ [°]	O–C•••C–O [°]	CH_2_–CH_2_–N_sp3_•••N_sp3_–CH_2_–CH_2_ [°]

empty	–	–	–	59.2	−41.0	79.9	−43.9
Li^+^	2.37, 2.44	2.82	3.42	−12.4	−97.0	58.0	−94.1
Na^+^	2.78, 2.79	2.84	2.89	23.8	−75.9	58.2	−77.0
K^+^	2.91, 2.93	2.96	2.84	34.6	−59.6	62.7	−61.9
Rb^+^	2.98, 3.00	3.04	2.90	43.7	−46.4	68.0	−48.7
Cs^+^	3.08, 3.09	3.11	2.98	53.7	−36.8	77.1	−38.6
Be^2+^ (*C_1_*)	1.69, 1.81, 1.77, 3.25	1.90, 4.48	3.62, 4.84	−42.9, −0.3	−102.0, −101.2	61.1	−96.5
Be^2+^	1.82, 1.89	2.92	4.17	−10.1	−114.5	55.8	−96.5
Mg^2+^ (*C_1_*)	2.41, 2.32, 2.34, 2.50	2.97, 2.62	2.31, 2.55	−20.1 −21.4	−109.2, −107.6	47.7	−106.6
Mg^2+^	2.28, 2.33	2.65	2.34	−10.4	−109.1	51.6	−104.3
Ca^2+^	2.69, 2.70	2.75	2.69	−10.5	−94.0	50.0	−95.9
Sr^2+^	2.81, 2.82	2.84	2.77	16.1	−76.9	52.5	−78.5
Ba^2+^	2.91, 2.92	2.96	2.84	28.4	−57.2	56.7	−59.5
Al^3+^	2.04, 2.08	2.34	3.83	−5.6	−126.2	48.4	−114.0
Ga^3+^	2.07, 2.12	2.42	3.77	−6.0	−123.2	48.2	−112.0
In^3+^	2.34, 2.37	2.64	2.56	−17.2	−121.7	45.8	−122.5
Tl^3+^	2.51, 2.53	2.65	2.82	−12.6	−109.0	50.5	−109.1

**Table 3:** Energy contributions (kcal/mol) to the complexation energy for [M 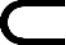 2.2.bpy]^m+^ (RB3LYP/LANL2DZp).

metal ions	Δ*E*_tot_	ΔZPE	complexation energy

Li^+^	23.0	−5.8	17.2
Li^+^(*C_1_*)	23.1	−5.9	17.2
Na^+^	9.6	−5.1	4.5
K^+^	1.8	−5.4	−3.6
Rb^+^	−7.3	−5.5	1.8
Cs^+^	18.5	−4.9	13.6
Be^2+^	6.7	−9.6	−3.1
Be^2+^(*C_1_*)	−11.9	−10.1	−22.0
Mg^2+^(*C_1_*)	9.1	−9.0	0.1
Mg^2+^(T.S.)	10.7	−9.0	1.7
Ca^2+^	−14.1	−8.3	−22.3
Sr^2+^	−18.4	−8.0	−26.4
Ba^2+^	−19.1	−7.0	−26.1
Al^3+^	−74.3	−19.2	−93.5
Ga^3+^(T.S.)	−62.9	−18.1	−81.0
Ga^3+^(*C_1_*)	−83.1	−18.0	−101.1
In^3+^	−75.9	−17.0	−92.9
Tl^3+^	−111.2	−15.9	−127.1

**Table 4:** Energy contributions (kcal/mol) to the complexation energy for [M 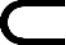 2.bpy.bpy]^m+^ (RB3LYP/LANL2DZp).

metal ions	Δ*E*_tot_	ΔZPE	complexation energy

Li^+^	8.6	−6.2	2.4
Na^+^	−3.7	−5.2	−8.9
K^+^	−8.9	−5.3	−14.2
Rb^+^	−1.2	−5.5	−6.7
Cs^+^	13.2	−5.1	8.1
Be^2+^	−20.0	−11.1	−31.1
Be^2+^(*C_1_*)	−33.5	−10.4	−43.9
Mg^2+^	−16.4	−9.5	−25.9
Mg^2+^(*C_1_*)	−20.0	−9.4	−29.4
Ca^2+^	−36.4	−8.7	−45.1
Sr^2+^	−38.2	−8.2	−46.4
Ba^2+^	−36.2	−7.1	−43.3
Al^3+^	−97.6	−11.6	−109.2
Ga^3+^	−111.0	−10.9	−121.9
In^3+^	−115.0	−9.4	−124.4
Tl^3+^	−150.1	−8.0	−158.1

Whereas [2.2.2] [2], [bpy.bpy.bpy] and [phen.phen.phen] [4] demonstrate *D_3_* symmetry, the cryptands studied in this work, [2.2.bpy] and [2.bpy.bpy], are mostly *C_2_* symmetric, like the arrangement found for [2.2.phen] and [2.phen.phen] [1]. For this reason [2.2.bpy] and [2.bpy.bpy] are able to host even small cations such as Be^2+^, Al^3+^, Ga^3+^ and Li^+^ effectively. They mostly prefer coordination to the nitrogen donor atoms, while for the larger ions the interaction with the oxygen donors also plays an important role.

**Figure 5:**
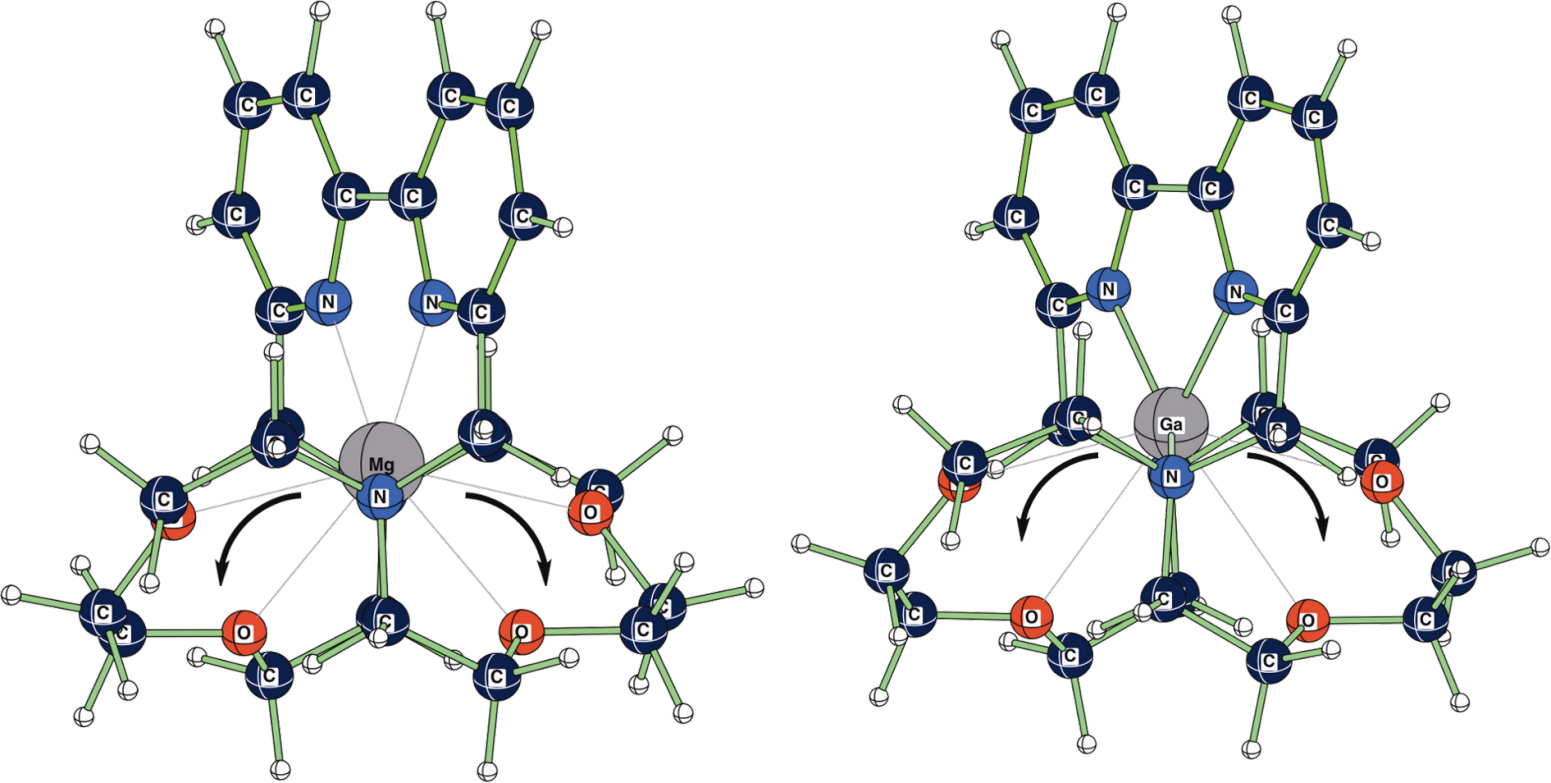
Calculated (RB3LYP/LANL2DZp) transition state structures (*C_2_*) for [Mg 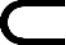 2.2.bpy]^2+^ and [Ga 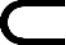 2.2.bpy]^3+^, showing the displacement vector for the imaginary frequency.

Except for [Mg 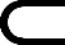 2.2.bpy]^2+^ and [Ga 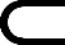 2.2.bpy]^3+^, all structures are local minima on the potential hypersurface, as noted in Table 3. The *C_2_* structures of [Mg 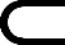 2.2.bpy]^2+^ and [Ga 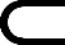 2.2.bpy]^3+^ are transition states for the movement of the metal ion inside the cavity of [2.2.bpy], as shown in Figure 5.

This motion leads from one glycol molecular bar to the other, bringing the cation closer to the O donor atoms, hence supporting the coordination. The barrier for this movement lies at 1.6 kcal/mol and 20.1 kcal/mol, respectively. The high value for the movement of Ga^3+^ inside the [2.2.bpy] results from the very effective complexation of earth metal ions by cryptands compared with single solvent molecules, e.g., H_2_O or NH_3_, and hence, a large amount of released free energy.

The presented work was initiated with the objective to systematically study the selective complexation of alkali, alkaline-earth and earth metal cations by [2.2.bpy] and [2.bpy.bpy] cryptands. In general, the prediction of a favorable complexation can be made based on two characteristics, viz., bond distances and energies of model reactions, as was shown in previous contributions from our group [1–4]. A comparison of the bond distances between the donor atoms and the metal cation complexed endohedrally by the cryptand or by the solvent molecules, e.g., pyridine or water, can be drawn. This method only provides reliable results if the donor atoms that coordinate to the metal center are the same and in an equal hybridization state in both cases. Therefore, we will compare the distances obtained in this work against [M(pyridine)*_n_*]^m+^ and [M(NH_3_)*_n_*]^m+^ (*n* = 4 for Li^+^ and Be^2+^ and 6 for all others), see Table 5, and against [M(H_2_O)*_n_*]^m+^ (*n* = 4 and 6 for Li^+^ and Be^2+^ and 6 for all others), see Table 6.

**Table 5:** Calculated distances (in Å) of the metal–donor interactions in [M(pyridine)*_n_*]^m+^ and [M(NH_3_)*_n_*]^m+^.

complex	M–N [Å]	symmetry	complex	M–N [Å]	symmetry

[Li(pyridine)_4_]^+^	2.07	*S_4_*	[Li(NH_3_)_4_]^+^	2.13	*T*
[Na(pyridine)_6_]^+^	2.62	*T_h_*	[Na(NH_3_)_6_]^+^	2.67	*C_2h_*
[K(pyridine)_6_]^+^	2.95	*C_i_*	[K(NH_3_)_6_]^+^	3.01	*C_2h_*
[Rb(pyridine)_6_]^+^	3.16	*C_i_*	[Rb(NH_3_)_6_]^+^	3.21	*C_2h_*
[Cs(pyridine)_6_]^+^	3.39	*C_i_*	[Cs(NH_3_)_6_]^+^	3.45	*C_2h_*
[Be(pyridine)_4_]^2+^	1.75	*S_4_*	[Be(NH_3_)_4_]^2+^	1.77	*T_d_*
[Mg(pyridine)_6_]^2+^	2.31	*T_h_*	[Mg(NH_3_)_6_]^2+^	2.29	*C_2h_*
[Ca(pyridine)_6_]^2+^	2.61	*T_h_*	[Ca(NH_3_)_6_]^2+^	2.63	*C_2h_*
[Sr(pyridine)_6_]^2+^	2.75	*T_h_*	[Sr(NH_3_)_6_]^2+^	2.80	*C_2h_*
[Ba(pyridine)_6_]^2+^	2.95	*C_i_*	[Ba(NH_3_)_6_]^2+^	3.00	*C_2h_*
[Al(pyridine)_6_]^3+^	2.15	*T_h_*	[Al(NH_3_)_6_]^3+^	2.12	*C_2_*
[Ga(pyridine)_6_]^3+^	2.18	*T_h_*	[Ga(NH_3_)_6_]^3+^	2.15	*C_2_*
[In(pyridine)_6_]^3+^	2.31	*T_h_*	[In(NH_3_)_6_]^3+^	2.31	*C_2_*
[Tl(pyridine)_6_]^3+^	2.44	*T_h_*	[Tl(NH_3_)_6_]^3+^	2.46	*C_2_*

**Table 6:** Calculated distances (in Å) of the metal–donor interactions in [M(H_2_O)*_n_*]^m+^.

complex	M-O [Å]	symmetry

^a^[Li(H_2_O)_6_]^+^ [Li(H_2_O)_4_]^+^	2.11 1.95	*T_h_* *C_2_*
^b^[Na(H_2_O)_6_]^+^	2.40	*T_h_*
[K(H_2_O)_6_]^+^	2.76	*T_h_*
[Rb(H_2_O)_6_]^+^	2.97	*T_h_*
[Cs(H_2_O)_6_]^+^	3.20	*T_h_*
[Be(H_2_O)_6_]^2+^ [Be(H_2_O)_4_]^2+^	1.85 1.65	*T_h_* *C_2_*
[Mg(H_2_O)_6_]^2+^	2.10	*T_h_*
[Ca(H_2_O)_6_]^2+^	2.43	*T_h_*
[Sr(H_2_O)_6_]^2+^	2.60	*T_h_*
[Ba(H_2_O)_6_]^2+^	2.80	*T_h_*
[Al(H_2_O)_6_]^3+^	1.96	*T_h_*
[Ga(H_2_O)_6_]^3+^	1.99	*T_h_*
[In(H_2_O)_6_]^3+^	2.14	*T_h_*
[Tl(H_2_O)_6_]^3+^	2.29	*T_h_*

^a,b^See [62].

**Figure 6:**
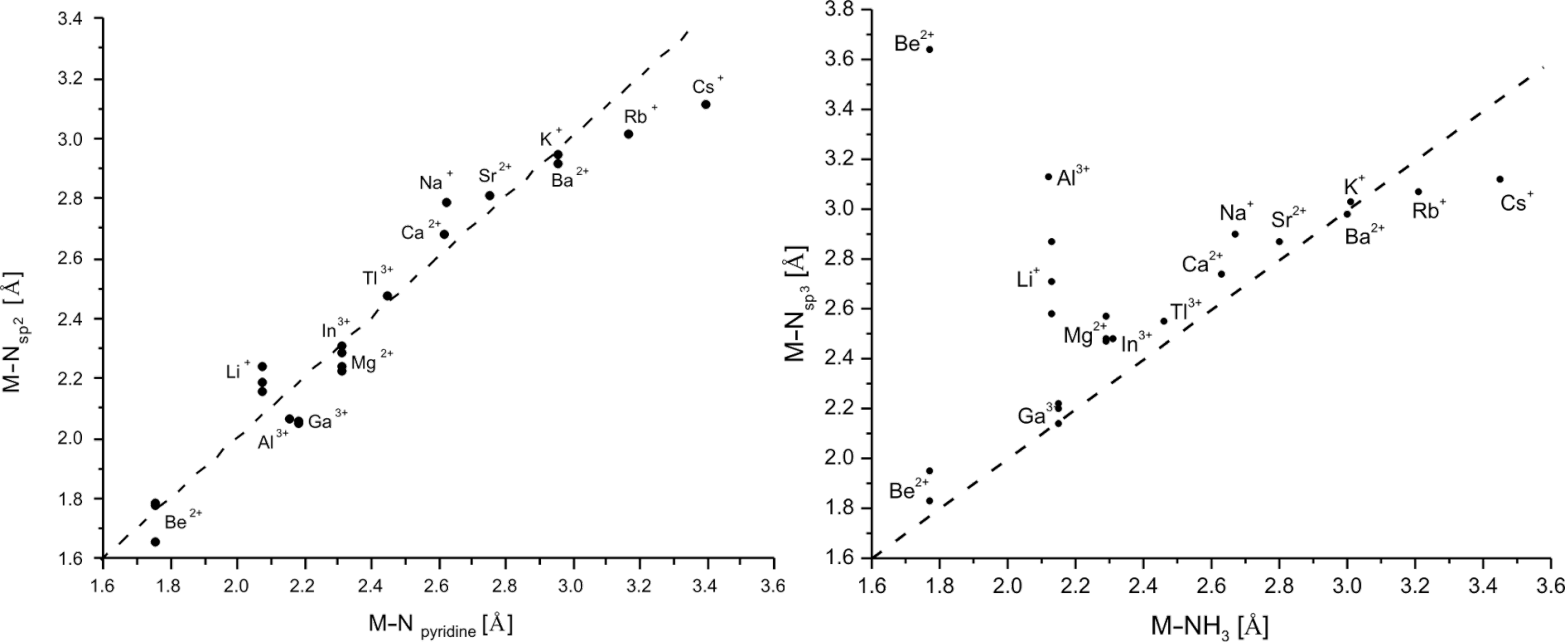
Comparison of the calculated (RB3LYP/LANL2DZp) M–N_pyridine_/M–N_sp2_ and M–NH_3_/M–N_sp3_ coordinating distances for [2.2.bpy] (dashed line: bisecting line; for the data see Table 7).

**Figure 7:**
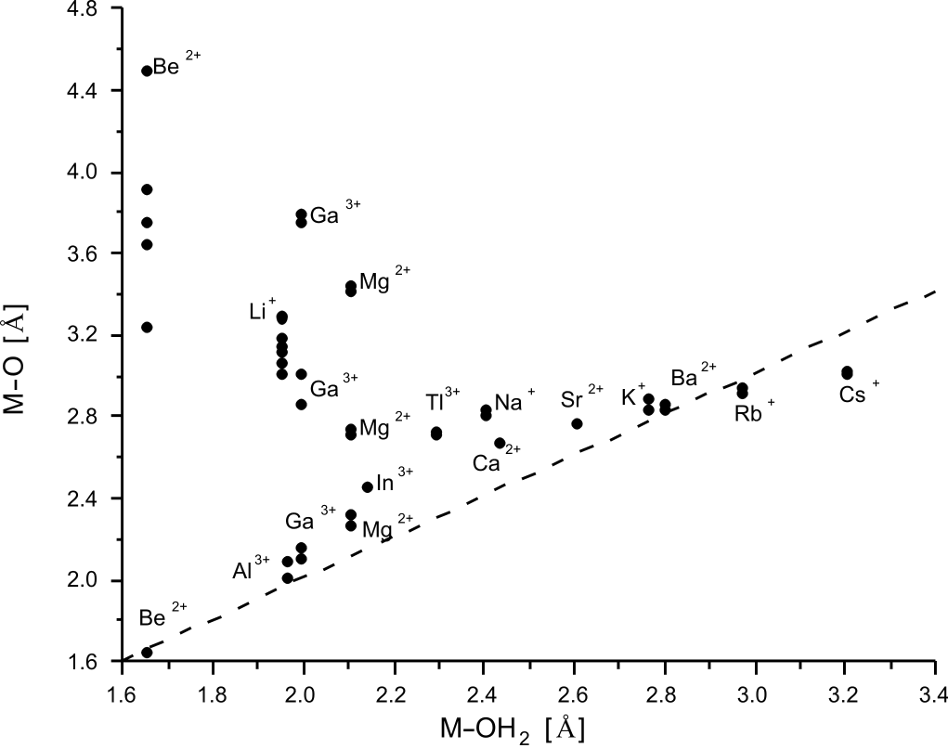
Comparison of the calculated (RB3LYP/LANL2DZp) M–OH_2_ and M–O coordinating distances for [2.2.bpy] (dashed line: bisecting line; for the data see Table 7).

**Figure 8:**
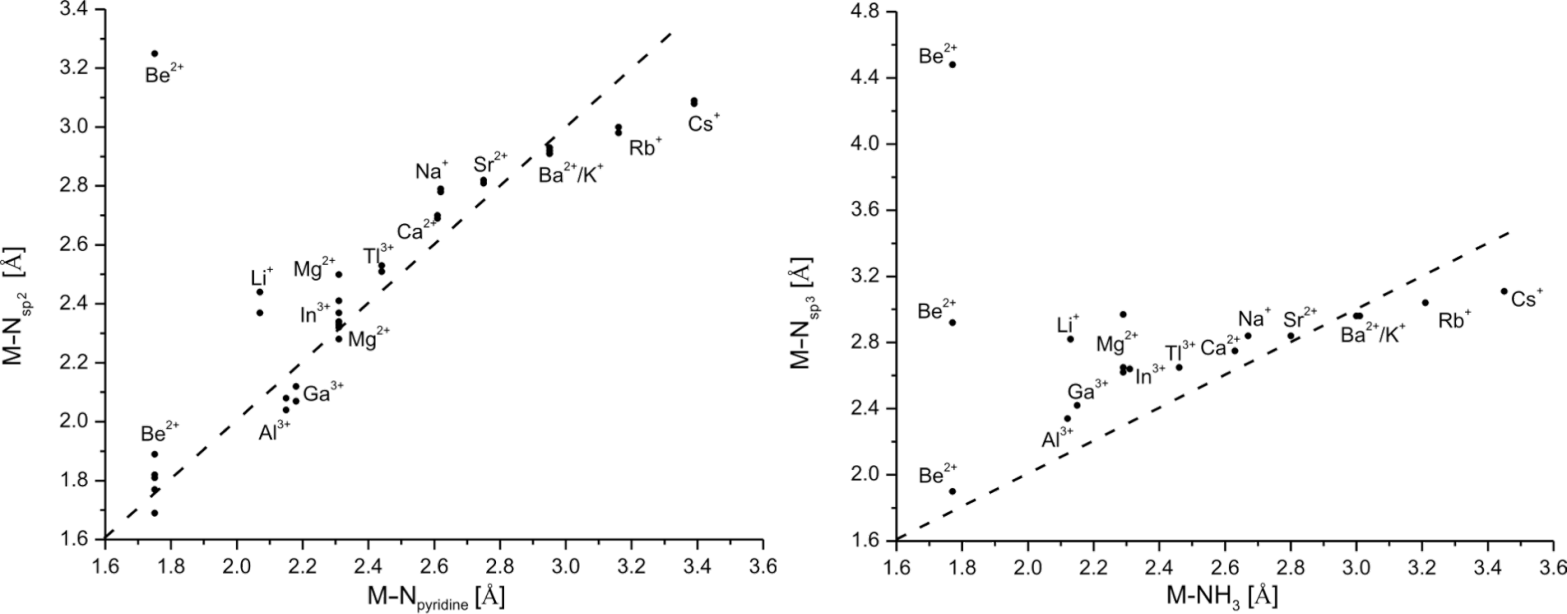
Comparison of the calculated (RB3LYP/LANL2DZp) M–N_pyridine_/M–N_sp2_ and M–NH_3_/M–N_sp3_ coordinating distances for [2.bpy.bpy] (dashed line: bisecting line; for the data see Table 8).

**Figure 9:**
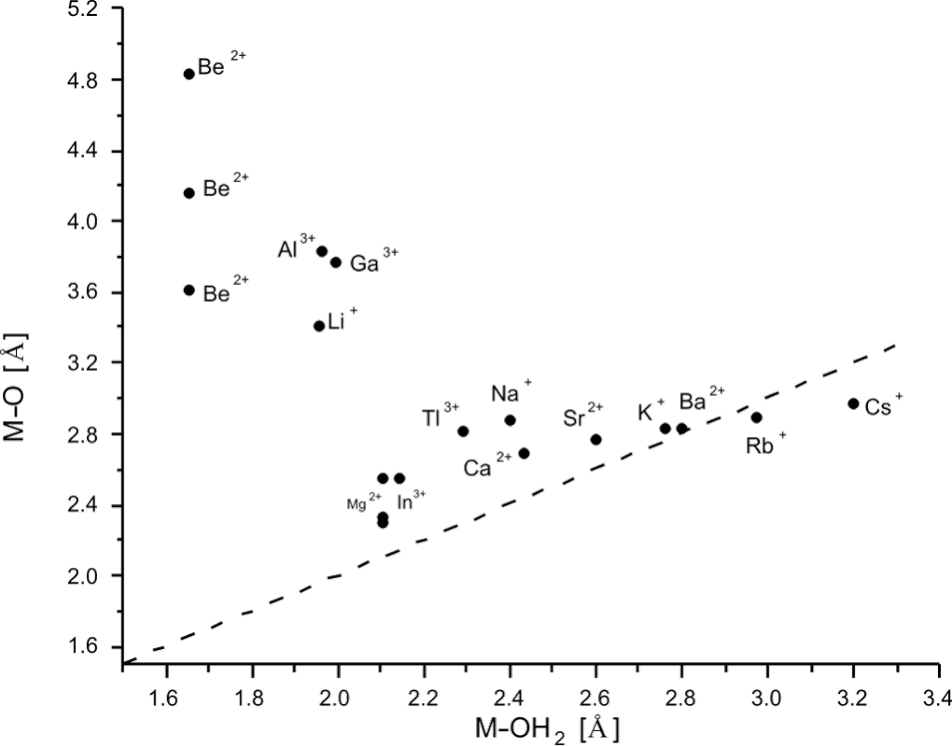
Comparison of the calculated (RB3LYP/LANL2DZp) M–OH_2_ and M–O coordinating distances for [2.bpy.bpy] (dashed line: bisecting line; for the data see Table 8).

A direct comparison of the calculated data for the metal donor atom bonds in cryptates and for solvated metal ions is given in Table 7 for [2.2.bpy] and Table 8 for [2.bpy.bpy]. To illustrate the situation more clearly, the results are presented in Figure 6 and Figure 7 for [2.2.bpy] and in Figure 8 and Figure 9 for [2.bpy.bpy], where bisecting lines point to the cases in which coordination is most likely to occur. The ions above the line are somewhat too small, whereas the ions below the line are too large for the studied cryptand.

**Table 7:** Comparison of the calculated distances (in Å) of the metal–donor interactions in [2.2.bpy] with [M(pyridine)*_n_*]^m+^, [M(NH_3_)*_n_*]^m+^ and [M(H_2_O)*_n_*]^m+^ (point groups are given in parenthesis, for most metal cations *C_2_* symmetry is adopted).

metal cation	M–N_sp2_	M–N_pyridine_	M–N_sp3_	M–NH_3_	M–O	M–OH_2_

Li^+^ (*C_1_*)	2.24, 2.16	2.07 (*S_4_*)	2.58, 2.87	2.13(*T*)	3.19, 3.01, 3.07, 3.29	1.95 (*C_2_*) 2.11 (*T_h_*)
Li^+^	2.19	2.07 (*S_4_*)	2.71	2.13 (*T*)	3.15, 3.12	1.95 (*C_2_*) 2.11 (*T_h_*)
Na^+^	2.79	2.62 (*T_h_*)	2.90	2.67 (*C_2h_*)	2.84, 2.81	2.40 (*T_h_*)
K^+^	2.95	2.95 (*C_i_*)	3.03	3.01 (*C_2h_*)	2.89, 2.84	2.76 (*T_h_*)
Rb^+^	3.02	3.16 (*C_i_*)	3.07	3.21 (*C_2h_*)	2.94, 2.92	2.97 (*T_h_*)
Cs^+^	3.12	3.39 (*C_i_*)	3.12	3.45 (*C_2h_*)	3.02, 3.03	3.20 (*T_h_*)
Be^2+^ (*C_1_*)	1.78, 1.66	1.75 (*S_4_*)	1.83, 3.64	1.77 (*T_d_*)	1.65, 3.76, 4.50, 3.92	1.65 (*C_2_*) 1.85 (*T_h_*)
Be^2+^	1.79	1.75 (*S_4_*)	1.95	1.77 (*T_d_*)	3.24, 3.65	1.65 (*C_2_*) 1.85 (*T_h_*)
Mg^2+^ (*C_1_*)	2.24, 2.23	2.31 (*T_h_*)	2.47, 2.57	2.29 (*C_2h_*)	2.33, 2.27, 3.42, 3.45	2.10 (*T_h_*)
Mg^2+^ (T.S.)	2.29	2.31 (*T_h_*)	2.48	2.29 (*C_2h_*)	2.71, 2.74	2.10 (*T_h_*)
Ca^2+^	2.68	2.61 (*T_h_*)	2.74	2.63 (*C_2h_*)	2.68, 2.68	2.43 (*T_h_*)
Sr^2+^	2.81	2.75 (*T_h_*)	2.87	2.80 (*C_2h_*)	2.77, 2.76	2.60 (*T_h_*)
Ba^2+^	2.92	2.95 (*C_i_*)	2.98	3.00 (*C_2h_*)	2.86, 2.84	2.80 (*T_h_*)
Al^3+^	2.07	2.15 (*T_h_*)	3.13	2.12 (*C_2_*)	2.02, 2.09	1.96 (*T_h_*)
Ga^3+^ (*C_1_*)	2.05, 2.05	2.18 (*T_h_*)	2.22, 2.20	2.15 (*C_2_*)	2.11, 2.16, 3.76, 3.80	1.99 (*T_h_*)
Ga^3+^ (T.S.)	2.06	2.18 (*T_h_*)	2.14	2.15 (*C_2_*)	2.86, 3.01	1.99 (*T_h_*)
In^3+^	2.31	2.31 (*T_h_*)	2.48	2.31 (*C_2_*)	2.46, 2,47	2.14 (*T_h_*)
Tl^3+^	2.48	2.44 (*T_h_*)	2.55	2.46 (*C_2_*)	2.71, 2.73	2.29 (*T_h_*)

**Table 8:** Comparison of the calculated distances (in Å) of the metal–donor interactions in [2.bpy.bpy] with [M(pyridine)*_n_*]^m+^, [M(NH_3_)*_n_*]^m+^ and [M(H_2_O)*_n_*]^m+^ (point groups are given in parenthesis, for most metal cations *C_2_* symmetry is adopted).

metal cation	M–N_sp2_	M–N_pyridine_	M–N_sp3_	M–NH_3_	M–O	M–OH_2_

Li^+^	2.37, 2.44	2.07 (*S_4_*)	2.82	2.13 (*T*)	3.42	1.95 (*C_2_*) 2.11 (*T_h_*)
Na^+^	2.78, 2.79	2.62 (*T_h_*)	2.84	2.67 (*C_2h_*)	2.89	2.40 (*T_h_*)
K^+^	2.91, 2.93	2.95 (*C_i_*)	2.96	3.01 (*C_2h_*)	2.84	2.76 (*T_h_*)
Rb^+^	2.98, 3.00	3.16 (*C_i_*)	3.04	3.21 (*C_2h_*)	2.90	2.97 (*T_h_*)
Cs^+^	3.08, 3.09	3.39 (*C_i_*)	3.11	3.45 (*C_2h_*)	2.98	3.20 (*T_h_*)
Be^2+^ (*C_1_*)	1.69, 1.81, 1.77, 3.25	1.75 (*S_4_*)	1.90, 4.48	1.77 (*T_d_*)	3.62, 4.84	1.65 (C_2_) 1.85 (*T_h_*)
Be^2+^	1.82, 1.89	1.75 (*S_4_*)	2.92	1.77 (*T_d_*)	4.17	1.65 (*C_2_*) 1.85 (*T_h_*)
Mg^2+^ (*C_1_*)	2.41, 2.32, 2.34, 2.50	2.31 (*T_h_*)	2.97, 2.62	2.29 (*C_2h_*)	2.31, 2.55	2.10 (*T_h_*)
Mg^2+^	2.28, 2.33	2.31 (*T_h_*)	2.65	2.29 (*C_2h_*)	2.34	2.10 (*T_h_*)
Ca^2+^	2.69, 2.70	2.61 (*T_h_*)	2.75	2.63 (*C_2h_*)	2.69	2.43 (*T_h_*)
Sr^2+^	2.81, 2.82	2.75 (*T_h_*)	2.84	2.80 (*C_2h_*)	2.77	2.60 (*T_h_*)
Ba^2+^	2.91, 2.92	2.95 (*C_i_*)	2.96	3.00 (*C_2h_*)	2.84	2.80 (*T_h_*)
Al^3+^	2.04, 2.08	2.15 (*T_h_*)	2.34	2.12 (*C_2_*)	3.83	1.96 (*T_h_*)
Ga^3+^	2.07, 2.12	2.18 (*T_h_*)	2.42	2.15 (*C_2_*)	3.77	1.99 (*T_h_*)
In^3+^	2.34, 2.37	2.31 (*T_h_*)	2.64	2.31 (*C_2_*)	2.56	2.14 (*T_h_*)
Tl^3+^	2.51, 2.53	2.44 (*T_h_*)	2.65	2.46 (*C_2_*)	2.82	2.29 (*T_h_*)

As depicted in Figure 6, the interaction of both N_sp2_ atoms of the bipyridine side of the [2.2.bpy] with all presented cations is essential. The bridgehead N_sp3_ atoms also play an important role for many of the studied ions, such as Ga^3+^, Tl^3+^, In^3+^, Ca^2+^, Sr^2+^, K^+^, Ba^2+^ and Rb^+^. However, for the larger (Na^+^, Cs^+^) and especially for the smaller ions (Mg^2+^, Al^3+^, Li^+^), the N_sp3_–M^m+^ interaction seems to be of lesser importance. The structure of [Be 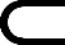 2.2.bpy]^2+^ presents a special case: in the energetically more stable *C_1_* symmetry (compared with *C_2_*, see later discussion), the Be^2+^ ion seems to be shifted towards one of the N_sp3_ atoms, as the calculated bond lengths differ significantly. Its fourth coordination site is occupied by one of the oxygen donor atoms.

The data set describing the interaction of oxygen donor atoms with the studied ions is shown in Figure 7. Only three cations lie on the bisecting line: the small Be^2+^ and the larger Ba^2+^ and Rb^+^, which is a good prerequisite for the coordination of the O-atoms to these cations. Further ions, such as Al^3+^, Ca^2+^, Sr^2+^, K^+^ and Cs^+^, are placed near the line, indicating that the proximity to the O-donors and a possible interaction still play an important role.

In the [Ga 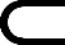 2.2.bpy]^3+(TS)^ and [Mg 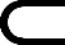 2.2.bpy]^2+(TS)^ cryptates, metal ions oscillate between both aliphatic di-ether chains, viz. between their oxygen donor atoms. Hence, half of the data points for Ga^3+^(*C_1_*) and Mg^2+^(*C_1_*) also lie close to the bisecting line, pointing to possible coordination. Finally, Li^+^, In^3+^, Tl^3+^ and Na^+^ as well as the residual data points for Be^2+^, Ga^3+^ and Mg^2+^ are positioned too far away from the line for the potential coordination to be relevant. At a first glance this finding seems to contradict the experimentally obtained results for Na^+^, but the solid-state structure of [Na 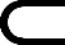 2.2.bpy]Br is somehow distorted (X-ray: *C_1_* symmetry compared to calculated *C_2_*) [61], to allow the cationic sodium center to interact with the available cryptand donor atoms and to get a better stabilization as in the applied weakly coordinating solvent acetonitrile [63–65].

It can be concluded that an interaction between Ca^2+^, Sr^2+^, K^+^, Ba^2+^ and Rb^+^ and all present donor atoms (N_sp2_, N_sp3_ and O) plays an important role. This can be explained, since especially the larger K^+^, Ba^2+^ and Rb^+^ ions prefer a higher coordination number than six [3]. They are also energetically favored (see further discussion) and seem to fit well into the cavity. In^3+^ and Tl^3+^ prefer the interaction with nitrogen donor atoms, completing the vacant positions with O-atoms. Among the earth metal ions Tl^3+^ is also energetically favored (see further discussion). Be^2+^ has a special position among the cations as described above, but it is still too small for the cryptand, as are also Li^+^, Mg^2+^, Al^3+^ and Ga^3+^. For Na^+^ and Cs^+^, the interaction with the bridgehead nitrogen appears not to be essential, more important are the connections to nitrogen atoms of the bipyridine side of the ligand and oxygen donor atoms. These cations also do not fit well into the cavity, being too small or too large, respectively.

As can be seen from Figure 8, the interaction with N_sp2_ atoms of the bipyridine site of [2.bpy.bpy] is significant for all studied ions, though the largest (Rb^+^, Cs^+^) deviate slightly from the general trend. A similar situation occurs for the bridgehead nitrogen atoms: most of the investigated cations are placed along the bisecting line, indicating an important role of the potential coordination. The large (Rb^+^, Cs^+^) and small (Li^+^, Mg^2+^(*C_1_*)) ions lie further away from the line, as this kind of interaction is less relevant for them. Be^2+^ again presents a special case, as it is shifted towards one of the N_sp3_ donor atoms and coordinates furthermore to three of the N_sp2_ atoms.

The interaction between oxygen donor atoms and the studied cations is less important in the case of [2.bpy.bpy], as there are six nitrogen donor atoms present to fill the coordination sphere. Even so, the bridgehead nitrogens are in some cases too far away for an effective interaction. As shown in Figure 9, only K^+^, Ba^2+^ and Rb^+^ lie on the bisecting line, while Mg^2+^, Ca^2+^, Sr^2+^ and Cs^+^ are placed near it. The residual cations, especially the small Be^2+^, Li^+^, Al^3+^ and Ga^3+^ are too far away for any kind of significant interaction.

Summing up, Ca^2+^, Sr^2+^, K^+^, Ba^2+^ and Rb^+^ fit best into the cavity of [2.bpy.bpy], as was the case for [2.2.bpy], though the smaller cations (Ca^2+^ and Sr^2+^) prefer the interaction with the nitrogen donor atoms and the larger cations (K^+^, Ba^2+^ and Rb^+^) tend to interact with the oxygen donors. The smaller cations are nested against the nitrogen atoms, though they do not fit well into the cavity. Among the earth metal ions, In^3+^ fits best, lying closer to the nitrogen donors and being also one of the energetically favored cations (Table 4).

According to the performed comparison of the bond lengths between metal cations and N/O donor atoms in cryptates with the same bond lengths of the solvated metal centers, both cavities have a size similar to [2.2.2] and prefer cations with larger radii in every one of the studied main groups. However, an additional energy consideration is necessary to provide detailed and more precise information about the favorable coordination layout of the investigated cryptands.

**Scheme 1:**
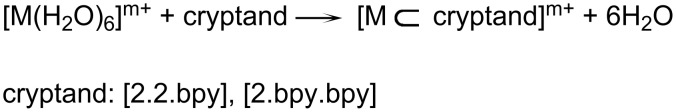
Model reaction.

**Figure 10:**
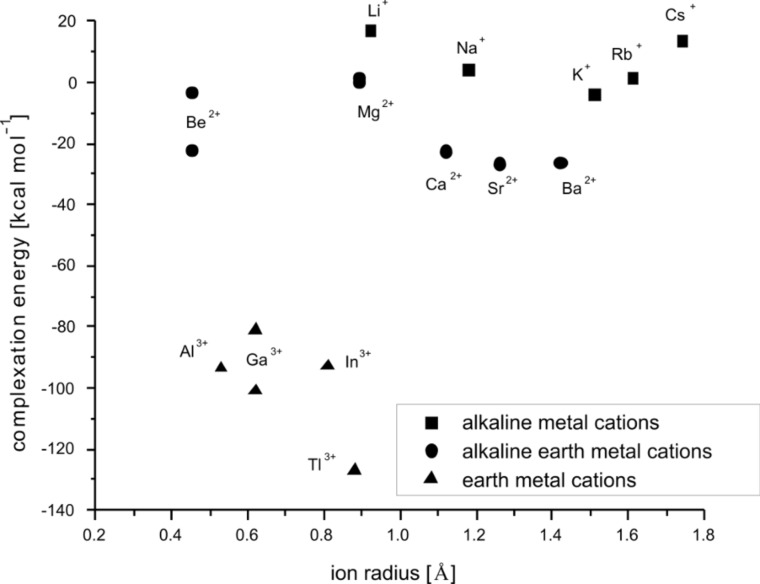
RB3LYP/LANL2DZp complexation energies for [M 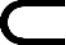 2.2.bpy]^m+^ according to Scheme 1, plotted against the ionic radius of M^m+^; for the data see Table 3.

**Figure 11:**
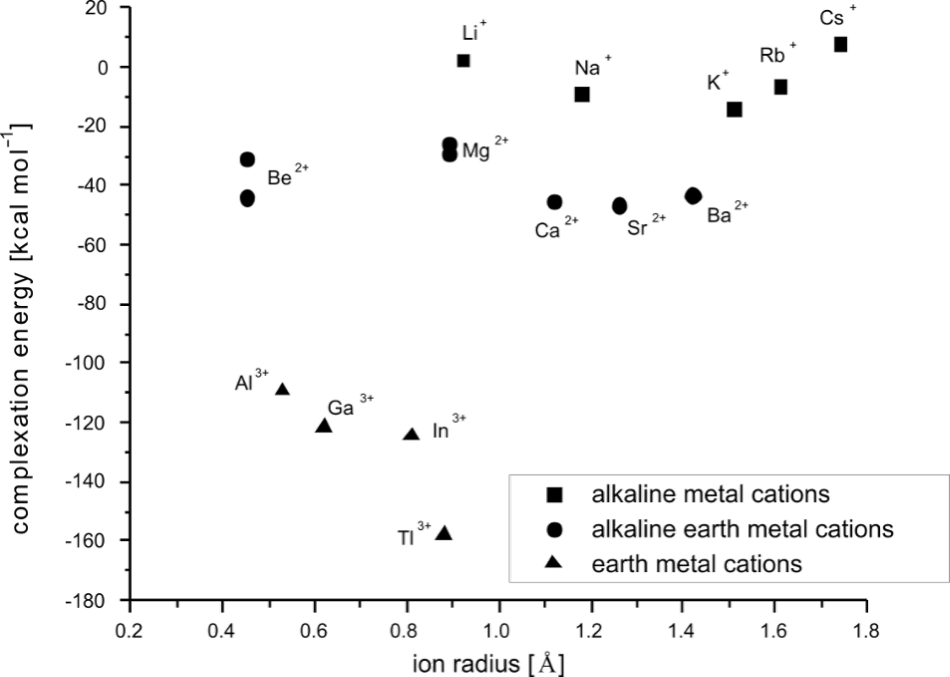
RB3LYP/LANL2DZp complexation energies for [M 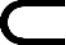 2.bpy.bpy]^m+^ according to Scheme 1, plotted against the ionic radius of M^m+^; for the data see Table 4.

Besides the structural evaluation of the computed systems, the examination of the energy of a model reaction, depicted in Scheme 1, provides valuable results, important for the prediction of the cryptand’s selectivity. All cations were calculated in the six-fold coordination environment, to maintain equal conditions for all studied systems. For the lithium [66] and beryllium [67] cations the preferable four-fold coordinated structures were found. They show additional water molecules, which do not interact directly with the metal center, but are held in the second coordination sphere by hydrogen bonds. However, the gas phase [Li(H_2_O)_6_]^+^ and [Be(H_2_O)_6_]^2+^ exist as local minima. The complexation energies computed in this way are shown in Table 3 and Table 4, and are plotted against the ionic radii, see Figure 10 and Figure 11.

The first and second places in the stability order of the endohedral complexes of [2.2.bpy] with the studied alkali metal ions are occupied by [K 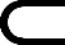 2.2.bpy]^+^ and [Rb 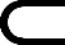 2.2.bpy]^+^. Among the examined alkaline earth ions the cryptates with Sr^2+^ and Ba^2+^ are the most stable and show an equal stability level. In the case of earth metal ions the most stable complex is formed for Tl^3+^, and this is in line with the high log *K*_1_ (9.4) of Tl^3+^ to form [Tl(bpy)]^3+^ [68–69]. Cryptand [2.bpy.bpy] also prefers to bind K^+^, this time followed by Na^+^; and Sr^2+^, followed by Ca^2+^. Finally the combination with Tl^3+^ yields the most stable cryptate. In general, the computed energies confirm our conclusions drawn from the bond lengths between the metal centers and donor atoms of the cryptands and/or solvent molecules. Both ligands [2.2.bpy] and [2.bpy.bpy] favor larger cations, indicating their large cavity size. A comparison of the preferred ion selectivity with the earlier reported results [2] demonstrates that both cryptands investigated in this work exhibit a hole size similar to that of [2.2.2], although [2.2.bpy] is somewhat larger than [2.bpy.bpy], which can be explained since the (bpy) part of the ligand is sterically more restrained and as a result the cryptand with two of them does not open up for larger guests.

The positive complexation energy for [Cs 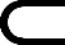 2.bpy.bpy]^+^, indicates that the Cs^+^ cation is too large for the cryptand cavity, while the positive energy value for [Li 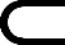 2.bpy.bpy]^+^, is a sign that the smallest alkali cation cannot be stabilized sufficiently. However, both values fit nicely in the trend. For all other ions studied here, the complexation with [2.bpy.bpy] results in negative stabilization energies.

In the case of [2.2.bpy], K^+^ is the only alkali cation for which complexation by the cryptand results in a negative stabilization energy, though the values for [Rb 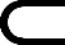 2.2.bpy]^+^ and [Na 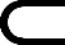 2.2.bpy]^+^ are very close, indicating ready coordination by the cryptand. The formation of cryptates with alkaline-earth and earth metal ions is again characterized by a negative stabilization energy, with the exception of [Mg 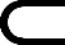 2.2.bpy]^2+(TS)^. The Be^2+^ ion presents a clear exception in both plots. As already mentioned, the beryllium dications are fourfold coordinated. In the case of [Be 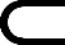 2.2.bpy]^2+^, the structure with *C_1_* symmetry is energetically more stable (−22.0 kcal/mol) than the one with *C_2_* symmetry (−3.1 kcal/mol). The beryllium ion is here coordinated by two N_sp2_, one N_sp3_ bridgehead and one O donor atom, so that an approximate tetrahedral coordination sphere with somewhat altered bonds (1.66 Å, 1.78 Å, 1.83 Å and 1.65 Å) compared to [Be(pyridine)_4_]^2+^ (1.75 Å), [Be(NH_3_)_4_]^2+^ (1.77 Å) and [Be(OH_2_)]^2+^ (1.65 Å) is formed. In the case of [Be 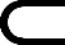 2.bpy.bpy], the structure with *C_1_* symmetry is favored again, though the difference in energy is not as large (−43.9 kcal/mol compared to −31.1 kcal/mol). The coordination of Be^2+^ in [Be 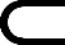 2.bpy.bpy]^2+^ is somewhat different, with the coordination sphere formed by three N_sp2_ and one N_sp3_ bridgehead donor atom. The bonds are further elongated compared to [Be 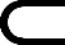 2.2.bpy]^2+^ and result in a distorted tetrahedron. Therefore, [Be 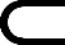 2.2.bpy]^2+^ and [Be 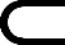 2.bpy.bpy]^2+^ can be considered as structures allowing appropriate coordination and stabilization in the gas phase, but in solution these structures will surely not be superior to solvated Be^2+^ and an empty cryptand. In general, the computed complexation energies allow conclusions about the stability order of the endohedral cryptate complexes and correlated with it about the ion selectivity of the respective cryptands, as will be explained below.

Earlier reports from our [1–4] and other [70] groups demonstrated that the cryptand does not remain unaltered throughout the complexation process; it twists in order to adjust for the optimal interaction with the metal cation. The effect of this twisting is observable also in the experimentally achieved solid-state structures. For example, [phen.phen.phen] has a very rigid structure, so the resulting average bond length between the metal center and the aromatic nitrogen donor atoms in [Na 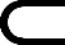 phen.phen.phen]^+^ are longer than the equivalent bonds in [Na 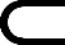 2.2.bpy]^+^ (av N_sp2_–Na = 2.70 Å [71] and av N_sp2_–Na = 2.61 Å [61], respectively). The [2.2.bpy] cryptand has namely two glycol-containing molecular arms, which can wrap flexibly around the cation, allowing closer proximity between the metal center and bipyridine ligand.

The increasing size of the guest ions is accompanied by a general enlargement of the metal–donor bond length, as can be concluded from the elucidation of the calculated distances (see Table 1 and Table 2). Apart from various contributions of the guest ions, the observed behavior is evidence for the possible flexibility of the studied cryptands. A proper flexibility requires an adaptable molecular moiety.

**Figure 12:**
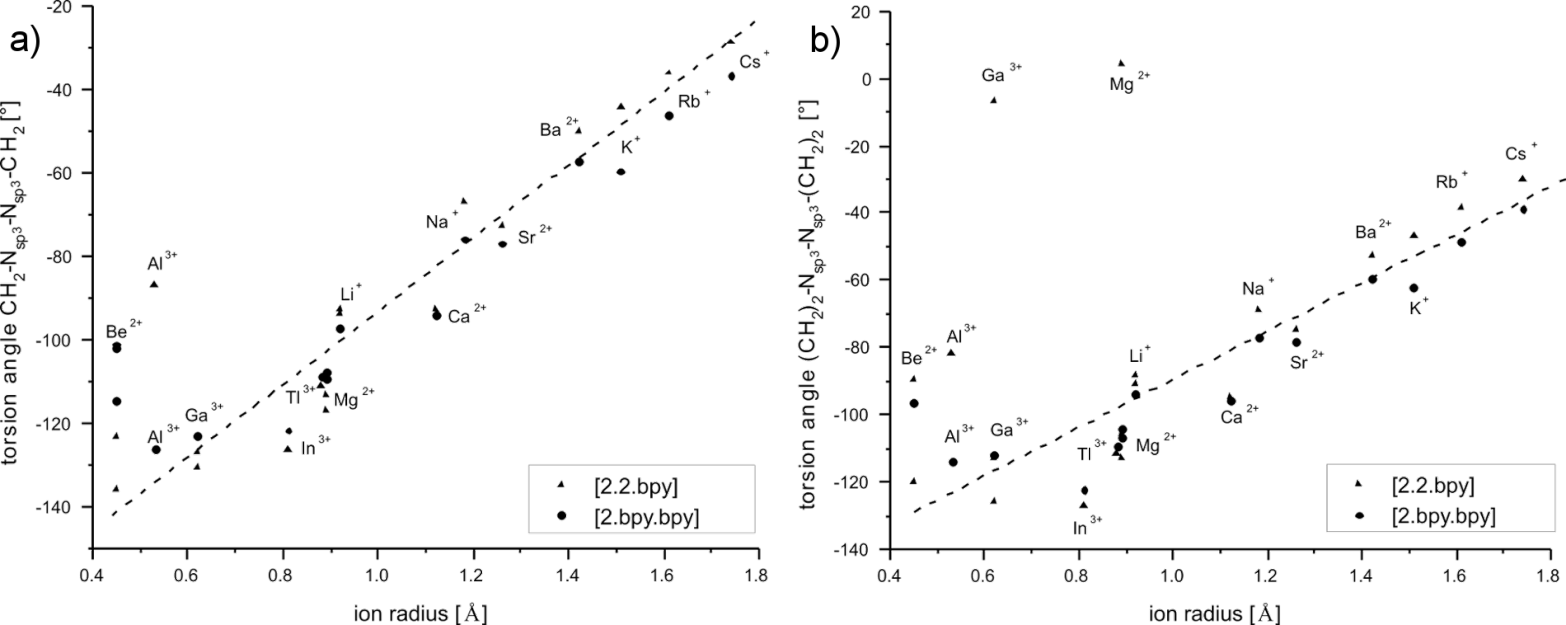
RB3LYP/LANL2DZp torsion angles CH_2_–N_sp3_–N_sp3_–CH_2_ (a) and (CH_2_)_2_–N_sp3_–N_sp3_–(CH_2_)_2_ (b) for [M 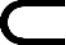 2.2.bpy]^m+^ and [M 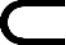 2.bpy.bpy]^m+^ plotted against the ionic radius of M^m+^ (dashed line represents the observed trend).

**Figure 13:**
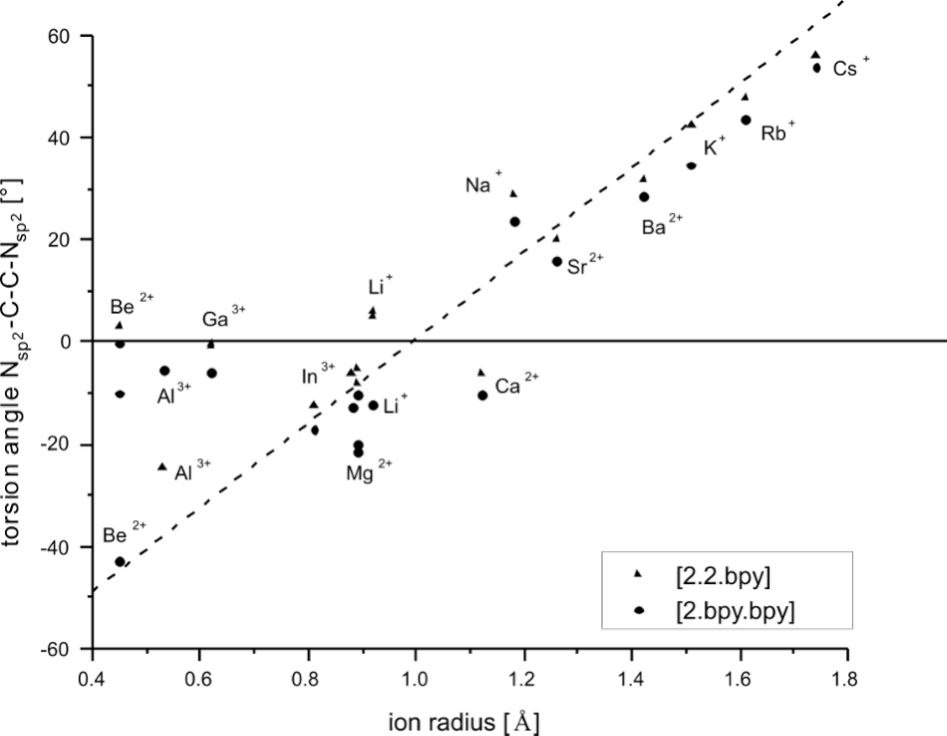
RB3LYP/LANL2DZp torsion angle N_sp2_–C–C–N_sp2_ for [M 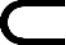 2.2.bpy]^m+^ and [M 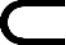 2.bpy.bpy]^m+^ plotted against the ionic radius of M^m+^ (dashed line represents the observed trend).

**Figure 14:**
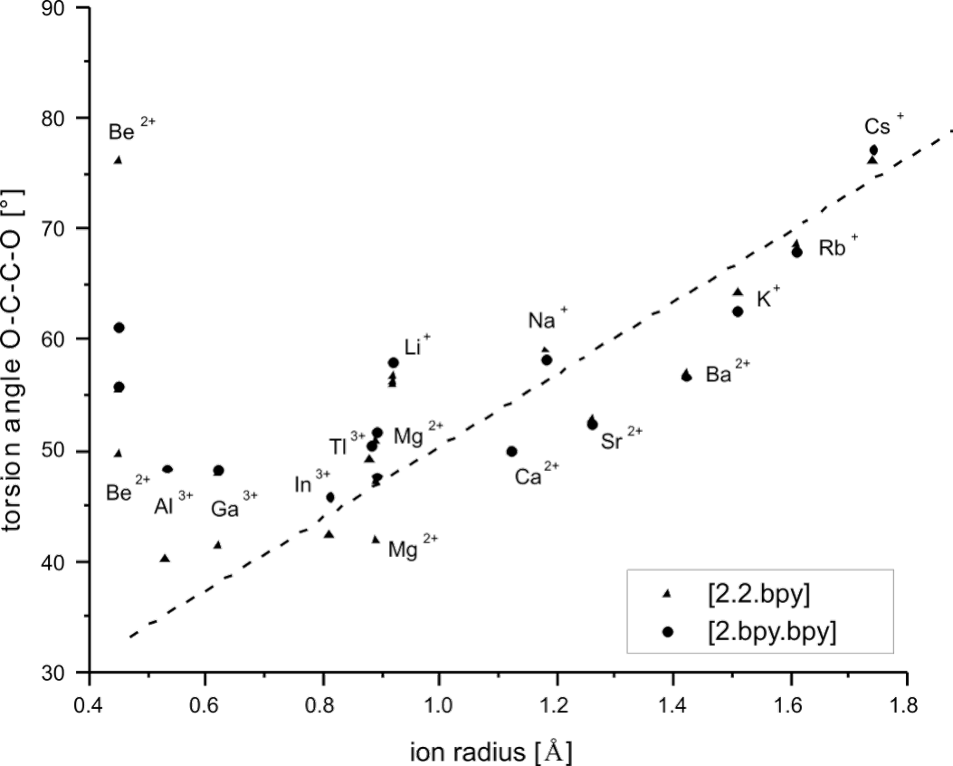
RB3LYP/LANL2DZp torsion angle O–C–C–O for [M 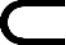 2.2.bpy]^m+^ and [M 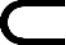 2.bpy.bpy]^m+^ plotted against the ionic radius of M^m+^ (dashed line represents the observed trend).

While [M 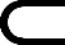 2.2.bpy]^m+^ has one molecular bar with N–CH_2_(pyridine) motifs adjacent to the N bridgeheads and a conformationally nearly unhampered C–C bond bridging the two pyridine rings and two molecular bars C_2_H_4_–O–C_2_H_4_–O–C_2_H_4_ adjacent to the N bridgehead atoms, the [M 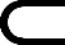 2.bpy.bpy]^m+^ has two of the bipyridine and one of the glycol building blocks. Descriptors for the twist and tilt of these moieties are the torsion angles such as CH_2_–N_sp3_–N_sp3_–CH_2_, N_sp2_–C–C–N_sp2_, (CH_2_)_2_–N_sp3_–N_sp3_–(CH_2_)_2_ and O–C–C–O. They all show a qualitative linear behavior that mainly depends on the size of the ion, see Figure 12, Figure 13 and Figure 14. The torsion angles CH_2_–N_sp3_–N_sp3_–CH_2_ and (CH_2_)_2_–N_sp3_–N_sp3_– (CH_2_)_2_ show very similar behavior, becoming more positive within the studied main groups, with the only exception presented by the earth metals, whose values are slightly scattered but still fit well in the general trend, see Figure 12. These angles cover the widest range in both host–guest systems, see Table 9.

**Table 9:** (Maximum) range of the calculated torsion angles.

	CH_2_–N_sp3_–N_sp3_–CH_2_	(CH_2_)_2_–N_sp3_–N_sp3_–(CH_2_)_2_	N_sp2_–C–C–N_sp2_	O–C–C–O

[2.2.bpy]	101.2°	96.9°	80.5°	35.9°
[2.bpy.bpy]	89.4°	83.9°	71.5°	31.3°

The illustrated occurrence evidences the importance of the possible alteration of the angles between the CH_2_ groups and the N_sp3_ bridgehead for optimal matching between the host and the guest molecules. The stereochemistry of all investigated cryptates is the same and can be described as λ, as the pointed angles are negative [3]. The only exception is the (CH_2_)_2_–N_sp3_–N_sp3_–(CH_2_)_2_ angle in [Mg 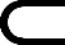 2.2.bpy]^2+(TS)^.

The N_sp2_–C–C–N_sp2_ torsion angle covers a somewhat smaller range than both angles at the bridgehead nitrogen atoms, as shown in Table 9, because of the greater rigidity of the connected pyridine rings compared to the aliphatic CH_2_ groups. However, the differences are much larger than in the case of [M 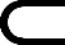 2.2.phen]^m+^ and [M 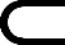 2.phen.phen]^m+^ [1], which is easy to understand since the polycyclic heteroaromatic phenantroline system is inflexible, while the conformationally nearly unhampered C–C bond bridging the two pyridine moieties allows more flexible arrangement of host and guest. The main exceptions of the linear trend are the cryptates with Be^2+^, Ga^3+^, Al^3+^ and Li^+^. As explained above, Be^2+^ cation presents a special case for both cryptates. In [Be 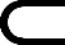 2.bpy.bpy]^2+^, the coordination sphere of the cation consists of four nitrogen atoms, viz., three coming from bpy-groups and the fourth being a bridgehead nitrogen. According to this, the cryptate in the *C_1_* symmetry, which is more stable than the *C_2_* symmetry, shows two N_sp2_–C–C–N_sp2_ twist angles: one of −0.3°, which lies above and one of −42.9°, which fits well in the general linear trend. In the case of [Be 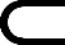 2.2.bpy]^2+^ the cation is again fourfold coordinated and shifted to one of the bridgehead nitrogen atoms, though this time one of the O-atoms is included in the coordination sphere. Because of its small radius, it apparently does not need to twist the chelating group as much as one would expect from the extrapolation of the other values. Similar behavior can be ascribed to other small cations: Al^3+^, Li^+^ and Ga^3+^, while [Ga 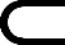 2.2.bpy]^3+^ in *C_2_* symmetry is present as a transition state, which can additionally contribute to the observed deviation. Throughout the series, cryptates change their stereochemistry. The twist angles for the smaller cations are negative and therefore the cryptates show a λ configuration; the larger ions cause positive angles and as a result the cryptates have a δ configuration.

Finally, the O–C–C–O torsion angles show the smallest range of all twist angles calculated here, though they are somewhat larger than the O–C–C–O angles found for [M 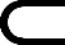 2.2.phen]^m+^ and [M 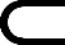 2.phen.phen]^m+^ [1]. The small values indicate that the twist of the molecules in this region is less important for the mutual adjustment of the host and guest. Apart from the exceptions described above, the coordination of the O atoms is mostly important for the larger cations for which Figure 14 shows a good linear trend, while the smaller ions deviate from it. In both cryptate series the angles are positive and become larger in the studied main groups. Hence, they show δ stereochemistry.

The four presented torsion angles describe the twist of the cryptand around the cations. The CH_2_–N_sp3_–N_sp3_–CH_2_ and (CH_2_)_2_–N_sp3_–N_sp3_–(CH_2_)_2_ angles lie in the middle of the molecule, pointing to the bipyridine and glycole building blocks, respectively. The N_sp2_–C–C–N_sp2_ and O–C–C–O angles lie above and under the middle of the molecule and the hosted cation at these molecular parts.

**Figure 15:**
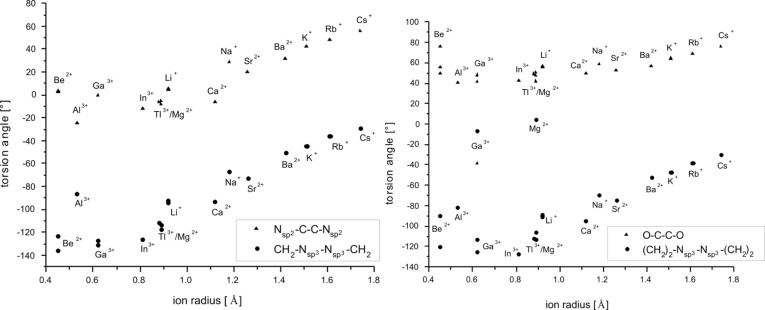
Reverse development of the calculated torsion angles on the respective cryptate sides for [2.2.bpy].

**Figure 16:**
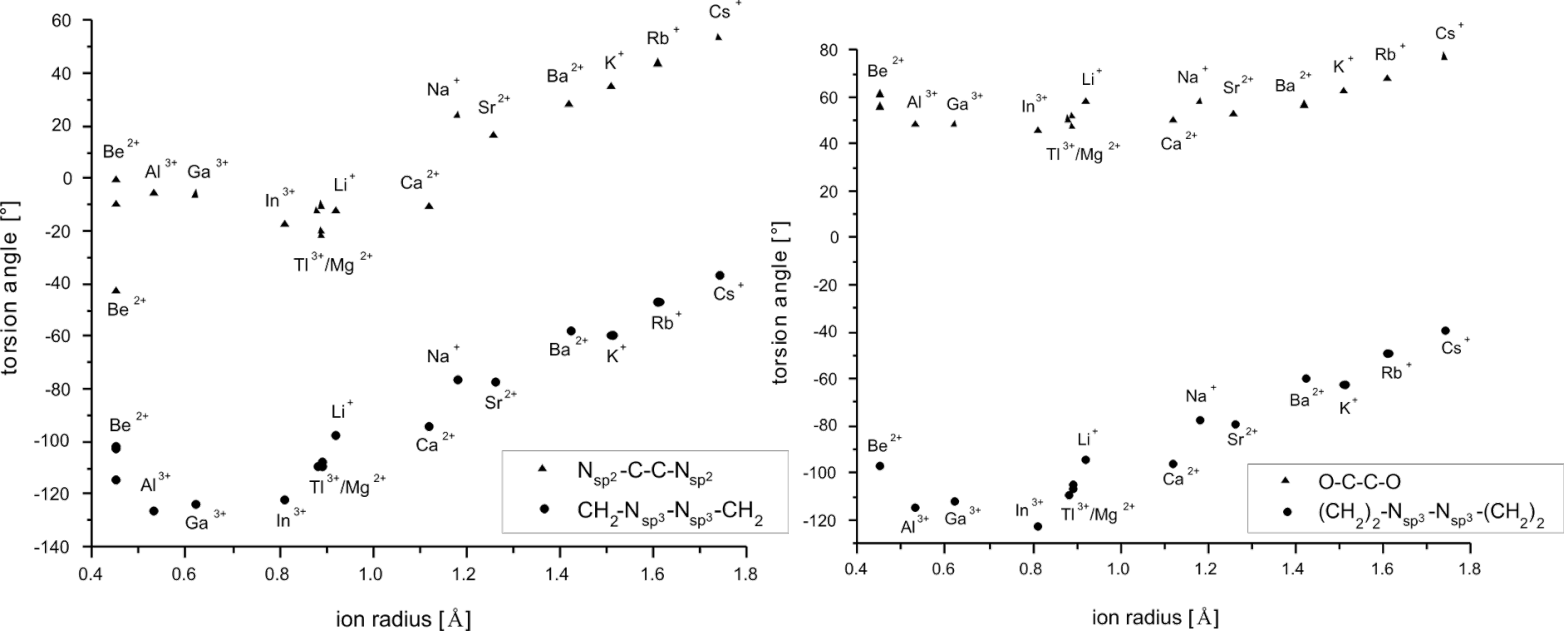
Reverse development of the calculated torsion angles on the respective cryptate sides for [2.bpy.bpy].

Comparison of the data given in Table 1 and Table 2 shows a mutual shift in the calculated values of the twist angles at the respective molecule halves. While the magnitude of the CH_2_–N_sp3_–N_sp3_–CH_2_ or (CH_2_)_2_–N_sp3_–N_sp3_–(CH_2_)_2_ angle becomes smaller, the N_sp2_–C–C–N_sp2_ or O–C–C–O angle is getting larger, for both cryptates alike, with the main exception presented by earth metals bound by [2.2.bpy], see Figure 15 and Figure 16. Hence, the cryptands coil around the hosted cations in order to get more tilted and closer to them.

As shown above, the analysis of the computed complexation energies plotted against the ionic radii allows conclusions to be made about the stability order of the studied endohedral cryptate complexes. Thereby the preferred selectivity of the alkaline earth ions plays the more important role, since the alkali cations are not very sensitive in this range, as the ion radius difference for Li^+^, Na^+^ and K^+^ is too large. The observed preference for the cation size can be correlated with the cavity size and, hence, with the selectivity of the cryptand. An overview of the preferred ion selectivity of several cryptand families recently studied in our group ([1–4] and the present contribution) is given in Table 10.

**Table 10:** Preferred ion selectivity of recently studied cryptands (no.: row number reflecting the cavity size and flexibility of the cryptand).

no.	host	preferred alkali ion	preferred alkaline earth ion

I	[2.2.2]	K > Rb	Ba > Sr
Ia	[N2N2N2]	K > Rb	Ba > Sr
II	[2.2.bpy]	K > Rb	Sr ≈ Ba
III	[2.2.phen]	K > Na	Sr > Ba
IVa	[2.bpy.bpy]	K > Na	Sr > Ca
IVb	[2.phen.phen]	K > Na	Sr > Ca
V	[bpy.bpy.bpy]	K > Na	Ca ≈ Sr, > Ba
VI	[phen.phen.phen]	Na > K	Ca ≈ Sr, > Ba
VII	[2.2.1]	Na > K	Ca > Sr
VIII	[2.1.1]	Li » Na	Mg > Ca
IXa	sarcophagine	Li » Na	Be > Mg
IXb	sepulchrate	Li » Na	Be > Mg

The cryptands are arranged according to their descending cavity size. The first in the row are [2.2.2] and [N2N2N2], which is easy to understand as they consist of conformationally nearly unhampered aliphatic molecular bars with oxygen or nitrogen donor atoms, respectively. These ligands are quite flexible, can arrange readily around the guests and, thus, can host larger cations. The list is continued by hybride cryptands, formed by a combination of [2.2.2] and [bpy.bpy.bpy] or [phen.phen.phen]. The substitution of every aliphatic di-ether chain by bipyridine or phenantroline molecular bars leads to reduced conformational flexibility and reduced cavity size. Thereby, the (phen) building block causes a stronger contraction of the cryptand hole, since the two pyridine rings are stiffened by a third, weaker aromatic six-membered ring (according to Clar’s rule [72–74]) as was explained above. Hence, the subsequent substitution by (bpy) and (phen) moieties reduces the size of the cations that the cryptand is able to bind. Notably, [2.bpy.bpy] and [2.phen.phen] have nearly the same cavity size, whereas [bpy.bpy.bpy] and [phen.phen.phen] are of a similar size and close to [2.2.1].

The next are [2.2.1] and [2.1.1], which can be derived from [2.2.2] by subsequent abstraction of one or two C_2_H_4_O moieties, respectively. The shorter aliphatic chains connecting the bridgehead nitrogen atoms are responsible for a considerable decrease in flexibility and cavity size of the cryptands, especially in the case of [2.1.1]. Sarcophagine and sepulchrate terminate the investigated series. Both of them can be derived from [N2N2N2]. In sarcophagine, the bridgehead nitrogen atoms are replaced by carbon atoms and in sepulchrate every aliphatic chain connecting the bridgehead nitrogen atoms is shortened by two CH_2_ units. These structural changes lead to more constitutional rigidity of the ligands and smaller cavity sizes, such that these two prefer the smallest cations of all cryptands examined throughout our study.

**Figure 17:**
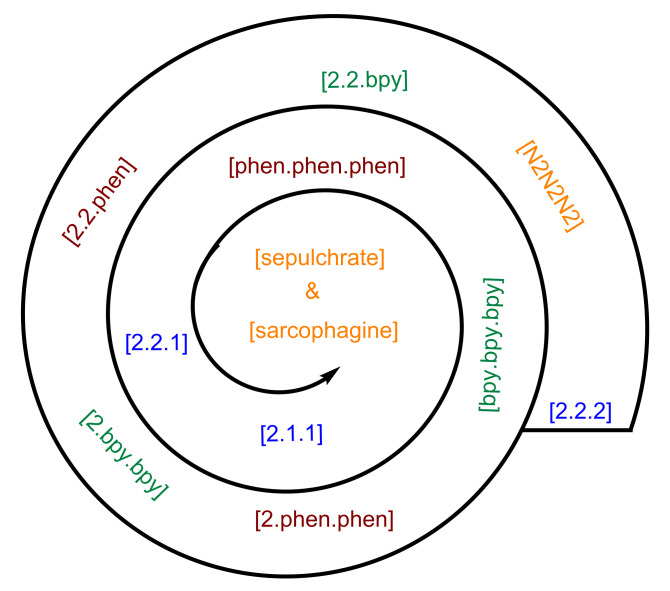
Trend in the preferred ion selectivity of the studied cryptands. Every cryptand family is distinguished by a different color.

To demonstrate our results more clearly, we arranged the cryptands schematically in a spiral shape shown in Figure 17, which not only includes already investigated ligands, but also provides space for further macromolecules that will be studied in future. Thereby, molecules of different size, viz., larger, smaller and those in between, will fit well into the given layout.

## Conclusion

According to our DFT-calculations, [2.2.bpy] and [2.bpy.bpy] have somewhat smaller cavities than [2.2.2]. Both cryptands prefer to bind Tl^3+^ as earth metal and K^+^ as alkali metal ions, although [2.2.bpy] favors Rb^+^ as the next best and [2.bpy.bpy] favors Na^+^. However, it is the selectivity of the alkaline-earth metal ions that is more significant if one wants to ascertain the cavity size of a cryptand. While [2.2.bpy] can bind Sr^2+^ and Ba^2+^ equally well, [2.bpy.bpy] forms more stable endohedral complexes with Sr^2+^ followed by Ca^2+^. The observed difference in the ion selectivity is an indication of a decreasing cavity size from [2.2.bpy] to [2.bpy.bpy].

The flexibility of the cryptands, which is important for selective host binding, is dominated by the flexibility of the CH_2_-units adjacent to the bridgehead nitrogen atoms. However, the contribution of the torsion angles of the bipyridine and glycole building blocks also plays an important role, the synchronous movement of the opposite molecule sides allows the cryptand to coil around the complexed cation.

The algebraic sign of the calculated angles allows the assignment of their stereochemistry. Throughout the series of all investigated cryptates, the molecular bars adjacent to the bridgehead nitrogen atoms show λ and the aliphatic di-ether chains δ stereochemistry, corresponding to negative and positive algebraic signs. Conversely, the steric configuration of the coordinating bipyridine moiety alternates depending on the size of the complexed metal ion.

## Experimental

### Quantum chemical methods

We performed B3LYP/LANL2DZp hybrid density functional calculations, i.e., with pseudo-potentials on the heavy elements and the valence basis set augmented with polarization functions [75–86]. During the optimization of the structures no other constraints than symmetry were applied. In addition, the resulting structures were characterized as minima, transition structures, etc., by computation of vibrational frequencies. The relative energies were corrected for zero-point vibrational energies (ZPE). We deliberately did not include any solvent model for the sake of comparability with earlier studies [1–4] and to exclude further approximations. The GAUSSIAN suite of programs was used [87].
